# 3-(12-Bromo­dodec­yl)-1,5-dimethyl-1*H*-1,5-benzodiazepine-2,4(3*H*,5*H*)-dione

**DOI:** 10.1107/S1600536810040134

**Published:** 2010-10-13

**Authors:** Rchida Dardouri, Fouad Ouazzani Chahdi, Natalie Saffon, El Mokhtar Essassi, Seik Weng Ng

**Affiliations:** a Laboratoire de Chimie Organique Hétérocyclique, Pôle de Compétences Pharmacochimie, Université Mohammed V-Agdal, BP 1014 Avenue Ibn Batout, Rabat, Morocco; b Service Commun Rayons-X FR2599, Université Paul Sabatier, Bâtiment 2R1, 118 route de Narbonne, Toulouse, France; c Department of Chemistry, University of Malaya, 50603 Kuala Lumpur, Malaysia

## Abstract

The seven-membered ring in the title compound, C_23_H_35_BrN_2_O_2_, adopts a boat-shaped conformation (with the C atoms of the fused-ring as the stern and the methine C atom as the prow). The bromo­dodecyl substituent occupies an equatorial position, with the dodecyl chain exhibiting an extended conformation. Weak inter­molecular C—H⋯O hydrogen bonding is present in the crystal structure.

## Related literature

For the crystal structure of 1,5-dimethyl-1,5-benzodiazepin-2,4-dione, see: Mondieig *et al.* (2005 ▶). For the structure of a similar compound, 3-(6-bromo­hex­yl)-1,5-dimethyl-1,5-benzodiazepine-2,4-dione, see: Dardouri *et al.* (2010 ▶).
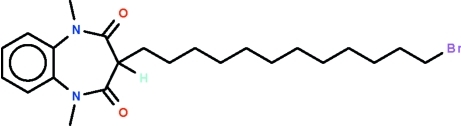


## Experimental

### 

#### Crystal data

C_23_H_35_BrN_2_O_2_
*M* *_r_* = 451.44
Monoclinic, 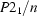

*a* = 7.5971 (1) Å
*b* = 10.5032 (2) Å
*c* = 28.7129 (5) Å
β = 95.213 (1)°
*V* = 2281.64 (7) Å^3^
*Z* = 4
Mo *K*α radiation
μ = 1.82 mm^−1^
*T* = 293 K
0.30 × 0.20 × 0.10 mm

#### Data collection

Bruker X8 APEXII diffractometer
Absorption correction: multi-scan (*SADABS*; Sheldrick, 1996 ▶) *T*_min_ = 0.611, *T*_max_ = 0.839
24581 measured reflections
6634 independent reflections
4370 reflections with *I* > 2σ(*I*)
*R*_int_ = 0.037

#### Refinement

*R*[*F* ^2^ > 2σ(*F* ^2^)] = 0.039
*wR*(*F* ^2^) = 0.099
*S* = 0.99
6634 reflections
255 parameters
H-atom parameters constrained
Δρ_max_ = 0.61 e Å^−3^
Δρ_min_ = −0.23 e Å^−3^

### 

Data collection: *APEX2* (Bruker, 2008 ▶); cell refinement: *SAINT* (Bruker, 2008 ▶); data reduction: *SAINT*; program(s) used to solve structure: *SHELXS97* (Sheldrick, 2008 ▶); program(s) used to refine structure: *SHELXL97* (Sheldrick, 2008 ▶); molecular graphics: *X-SEED* (Barbour, 2001 ▶); software used to prepare material for publication: *publCIF* (Westrip, 2010 ▶).

## Supplementary Material

Crystal structure: contains datablocks global, I. DOI: 10.1107/S1600536810040134/xu5050sup1.cif

Structure factors: contains datablocks I. DOI: 10.1107/S1600536810040134/xu5050Isup2.hkl

Additional supplementary materials:  crystallographic information; 3D view; checkCIF report

## Figures and Tables

**Table 1:** Hydrogen-bond geometry (Å, °)

*D*—H⋯*A*	*D*—H	H⋯*A*	*D*⋯*A*	*D*—H⋯*A*
C2—H2⋯O1^i^	0.93	2.52	3.424 (2)	164
C7—H7*B*⋯O2^ii^	0.96	2.40	3.340 (2)	166
C11—H11*C*⋯O1^ii^	0.96	2.48	3.407 (2)	164

Symmetry codes: (i) 
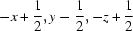
; (ii) 
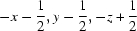
.

**Fig. 1.**
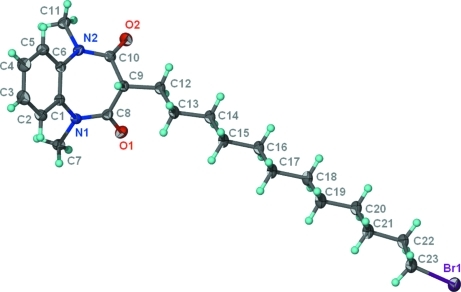
Thermal ellipsoid plot (Barbour, 2001) of C23H35BrN2O2 at the 50% probability level; hydrogen atoms are drawn as arbitrary radius.

## supplementary crystallographic information

### Comment

The methylene part of 1,5-dimethyl-1,5-benzodiazepine-2,4-dione is relatively acidic, and one proton can be abstracted by using potassium *t*-butoxide; the resulting carbanion can undergo a nucleophlilic subsitution with a dibromoalkane to form 3-substituted derivatives. In this study, the compound is reacted with 1,12-dibromododecane the title compound (Scheme I, Fig. 1).

### Experimental

To a solution of the potassium *t*-butoxide (0.42 g, 3.6 mmol) in DMF (15 ml) was added 1,5-dimethyl-1,5-benzodiazepine-2,4-dione (0.50 g, 2.4 mmol) and 1,12-dibromododecane (0.94 g, 2.88 mmol). Stirring was continued for 24 h. The reaction was monitored by thin layer chromatography. The mixture was filtered and the solution evaporated to give colorless crystals.

### Refinement

Carbon-bound H-atoms were placed in calculated positions (C—H 0.93–0.97 Å) and were included in the refinement in the riding model approximation, with *U*(H) set to 1.2–1.5*U*_eq_(C).

### Crystal data

**Table:**

C_23_H_35_BrN_2_O_2_	*F*(000) = 952
*M**_r_* = 451.44	*D*_x_ = 1.314 Mg m^−^^3^
Monoclinic, *P*2_1_/*n*	Mo *K*α radiation, λ = 0.71073 Å
Hall symbol: -P 2yn	Cell parameters from 5655 reflections
*a* = 7.5971 (1) Å	θ = 2.4–25.4°
*b* = 10.5032 (2) Å	µ = 1.82 mm^−^^1^
*c* = 28.7129 (5) Å	*T* = 293 K
β = 95.213 (1)°	Prism, colorless
*V* = 2281.64 (7) Å^3^	0.30 × 0.20 × 0.10 mm
*Z* = 4	

### Data collection

**Table:**

Bruker X8 APEXII diffractometer	6634 independent reflections
Radiation source: fine-focus sealed tube	4370 reflections with *I* > 2σ(*I*)
graphite	*R*_int_ = 0.037
φ and ω scans	θ_max_ = 30.0°, θ_min_ = 2.7°
Absorption correction: multi-scan (*SADABS*; Sheldrick, 1996)	*h* = −10→10
*T*_min_ = 0.611, *T*_max_ = 0.839	*k* = −14→12
24581 measured reflections	*l* = −40→40

### Refinement

**Table:**

Refinement on *F*^2^	Primary atom site location: structure-invariant direct methods
Least-squares matrix: full	Secondary atom site location: difference Fourier map
*R*[*F*^2^ > 2σ(*F*^2^)] = 0.039	Hydrogen site location: inferred from neighbouring sites
*wR*(*F*^2^) = 0.099	H-atom parameters constrained
*S* = 0.99	*w* = 1/[σ^2^(*F*_o_^2^) + (0.0518*P*)^2^] where *P* = (*F*_o_^2^ + 2*F*_c_^2^)/3
6634 reflections	(Δ/σ)_max_ = 0.001
255 parameters	Δρ_max_ = 0.61 e Å^−^^3^
0 restraints	Δρ_min_ = −0.23 e Å^−^^3^

### Fractional atomic coordinates and isotropic or equivalent isotropic displacement parameters (Å^2^)

**Table:**

	*x*	*y*	*z*	*U*_iso_*/*U*_eq_	
Br1	1.65169 (3)	1.558652 (19)	0.586300 (7)	0.04634 (9)	
N1	0.0090 (2)	0.57047 (12)	0.26467 (5)	0.0294 (3)	
N2	−0.32138 (19)	0.57806 (12)	0.30691 (5)	0.0291 (3)	
O1	0.08035 (17)	0.77902 (11)	0.26153 (4)	0.0368 (3)	
O2	−0.35919 (17)	0.78733 (11)	0.32234 (5)	0.0411 (3)	
C1	−0.0532 (2)	0.46396 (15)	0.28941 (6)	0.0276 (4)	
C2	0.0475 (2)	0.35221 (16)	0.29190 (7)	0.0350 (4)	
H2	0.1551	0.3497	0.2788	0.042*	
C3	−0.0126 (3)	0.24597 (17)	0.31369 (7)	0.0411 (5)	
H3	0.0549	0.1720	0.3154	0.049*	
C4	−0.1723 (3)	0.24865 (17)	0.33297 (7)	0.0407 (5)	
H4	−0.2131	0.1762	0.3472	0.049*	
C5	−0.2713 (3)	0.35867 (16)	0.33120 (7)	0.0347 (4)	
H5	−0.3781	0.3605	0.3447	0.042*	
C6	−0.2126 (2)	0.46761 (15)	0.30930 (6)	0.0266 (4)	
C7	0.0742 (3)	0.54706 (17)	0.21882 (6)	0.0355 (4)	
H7A	0.0659	0.6240	0.2007	0.053*	
H7B	0.0041	0.4819	0.2027	0.053*	
H7C	0.1953	0.5200	0.2231	0.053*	
C8	0.0180 (2)	0.69084 (15)	0.28235 (6)	0.0285 (4)	
C9	−0.0598 (2)	0.70942 (15)	0.32913 (6)	0.0273 (4)	
H9	−0.0165	0.6412	0.3505	0.033*	
C10	−0.2600 (2)	0.69690 (16)	0.31965 (6)	0.0292 (4)	
C11	−0.5119 (2)	0.56279 (17)	0.29472 (7)	0.0350 (4)	
H11A	−0.5592	0.6397	0.2805	0.053*	
H11B	−0.5690	0.5454	0.3225	0.053*	
H11C	−0.5320	0.4933	0.2732	0.053*	
C12	−0.0055 (2)	0.83761 (16)	0.35126 (7)	0.0327 (4)	
H12A	−0.0200	0.9037	0.3276	0.039*	
H12B	−0.0823	0.8576	0.3755	0.039*	
C13	0.1849 (2)	0.83595 (15)	0.37232 (6)	0.0329 (4)	
H13A	0.2597	0.8092	0.3485	0.039*	
H13B	0.1965	0.7732	0.3972	0.039*	
C14	0.2511 (2)	0.96386 (16)	0.39196 (7)	0.0328 (4)	
H14A	0.2437	1.0262	0.3669	0.039*	
H14B	0.1745	0.9921	0.4152	0.039*	
C15	0.4398 (3)	0.95858 (15)	0.41418 (7)	0.0335 (4)	
H15A	0.5142	0.9229	0.3918	0.040*	
H15B	0.4446	0.9015	0.4408	0.040*	
C16	0.5151 (3)	1.08722 (16)	0.43040 (7)	0.0348 (4)	
H16A	0.5133	1.1439	0.4037	0.042*	
H16B	0.4398	1.1239	0.4524	0.042*	
C17	0.7028 (3)	1.07876 (15)	0.45343 (7)	0.0354 (4)	
H17A	0.7779	1.0431	0.4312	0.043*	
H17B	0.7045	1.0206	0.4797	0.043*	
C18	0.7796 (3)	1.20563 (16)	0.47064 (7)	0.0365 (4)	
H18A	0.7819	1.2629	0.4442	0.044*	
H18B	0.7025	1.2427	0.4921	0.044*	
C19	0.9648 (3)	1.19565 (16)	0.49503 (7)	0.0383 (4)	
H19A	1.0418	1.1593	0.4734	0.046*	
H19B	0.9625	1.1373	0.5212	0.046*	
C20	1.0436 (3)	1.32136 (16)	0.51308 (7)	0.0362 (4)	
H20A	1.0439	1.3808	0.4872	0.043*	
H20B	0.9696	1.3568	0.5357	0.043*	
C21	1.2304 (3)	1.30689 (16)	0.53584 (7)	0.0370 (4)	
H21A	1.3013	1.2651	0.5139	0.044*	
H21B	1.2278	1.2516	0.5628	0.044*	
C22	1.3211 (3)	1.43160 (15)	0.55161 (7)	0.0365 (4)	
H22A	1.3216	1.4894	0.5253	0.044*	
H22B	1.2569	1.4718	0.5753	0.044*	
C23	1.5070 (3)	1.40468 (18)	0.57104 (7)	0.0409 (5)	
H23A	1.5643	1.3542	0.5485	0.049*	
H23B	1.5038	1.3539	0.5992	0.049*	

### Atomic displacement parameters (Å^2^)

**Table:**

	*U*^11^	*U*^22^	*U*^33^	*U*^12^	*U*^13^	*U*^23^
Br1	0.05160 (15)	0.04100 (12)	0.04792 (14)	−0.01138 (9)	0.01260 (10)	−0.00678 (9)
N1	0.0297 (8)	0.0275 (7)	0.0323 (8)	−0.0030 (6)	0.0092 (6)	−0.0026 (6)
N2	0.0229 (7)	0.0252 (7)	0.0392 (8)	0.0003 (5)	0.0030 (6)	−0.0004 (6)
O1	0.0387 (7)	0.0305 (6)	0.0421 (7)	−0.0073 (5)	0.0089 (6)	0.0033 (5)
O2	0.0327 (7)	0.0307 (7)	0.0598 (9)	0.0082 (6)	0.0037 (6)	−0.0064 (6)
C1	0.0281 (9)	0.0249 (8)	0.0300 (9)	−0.0010 (7)	0.0030 (7)	−0.0014 (6)
C2	0.0304 (10)	0.0325 (9)	0.0428 (11)	0.0046 (7)	0.0072 (8)	−0.0037 (8)
C3	0.0466 (12)	0.0276 (9)	0.0492 (12)	0.0100 (8)	0.0057 (9)	0.0017 (8)
C4	0.0507 (12)	0.0294 (9)	0.0433 (11)	−0.0005 (8)	0.0114 (9)	0.0078 (8)
C5	0.0340 (10)	0.0311 (9)	0.0405 (10)	−0.0021 (7)	0.0116 (8)	0.0013 (7)
C6	0.0257 (9)	0.0238 (8)	0.0305 (9)	0.0006 (6)	0.0029 (7)	−0.0015 (6)
C7	0.0343 (10)	0.0403 (10)	0.0331 (10)	−0.0048 (8)	0.0097 (8)	−0.0036 (8)
C8	0.0230 (9)	0.0274 (8)	0.0348 (9)	−0.0007 (7)	0.0002 (7)	−0.0007 (7)
C9	0.0255 (9)	0.0239 (8)	0.0323 (9)	0.0008 (6)	0.0015 (7)	−0.0024 (6)
C10	0.0286 (9)	0.0275 (8)	0.0317 (9)	0.0023 (7)	0.0041 (7)	−0.0009 (7)
C11	0.0254 (9)	0.0361 (9)	0.0431 (11)	−0.0003 (8)	0.0002 (8)	−0.0007 (8)
C12	0.0322 (10)	0.0257 (8)	0.0402 (10)	0.0003 (7)	0.0031 (8)	−0.0054 (7)
C13	0.0360 (10)	0.0253 (8)	0.0371 (10)	−0.0006 (7)	0.0017 (8)	−0.0047 (7)
C14	0.0375 (10)	0.0258 (8)	0.0351 (10)	−0.0005 (7)	0.0034 (8)	−0.0030 (7)
C15	0.0396 (11)	0.0257 (9)	0.0345 (10)	−0.0019 (7)	0.0000 (8)	−0.0036 (7)
C16	0.0414 (11)	0.0252 (9)	0.0378 (10)	−0.0010 (7)	0.0038 (8)	−0.0046 (7)
C17	0.0426 (11)	0.0263 (9)	0.0366 (10)	−0.0045 (7)	−0.0009 (8)	−0.0050 (7)
C18	0.0429 (11)	0.0252 (9)	0.0412 (10)	−0.0027 (8)	0.0024 (9)	−0.0049 (7)
C19	0.0449 (11)	0.0287 (9)	0.0400 (11)	−0.0014 (8)	−0.0025 (9)	−0.0040 (8)
C20	0.0419 (11)	0.0281 (9)	0.0384 (10)	−0.0033 (8)	0.0028 (8)	−0.0039 (7)
C21	0.0454 (11)	0.0271 (9)	0.0377 (10)	−0.0014 (8)	−0.0006 (9)	−0.0021 (7)
C22	0.0435 (11)	0.0272 (9)	0.0384 (10)	−0.0023 (8)	0.0023 (9)	−0.0027 (7)
C23	0.0442 (12)	0.0322 (9)	0.0457 (11)	−0.0041 (8)	0.0003 (9)	−0.0067 (8)

### Geometric parameters (Å, °)

**Table:**

Br1—C23	1.9818 (19)	C13—C14	1.525 (2)
N1—C8	1.362 (2)	C13—H13A	0.9700
N1—C1	1.428 (2)	C13—H13B	0.9700
N1—C7	1.469 (2)	C14—C15	1.516 (3)
N2—C10	1.371 (2)	C14—H14A	0.9700
N2—C6	1.423 (2)	C14—H14B	0.9700
N2—C11	1.466 (2)	C15—C16	1.523 (2)
O1—C8	1.221 (2)	C15—H15A	0.9700
O2—C10	1.219 (2)	C15—H15B	0.9700
C1—C6	1.386 (2)	C16—C17	1.519 (3)
C1—C2	1.399 (2)	C16—H16A	0.9700
C2—C3	1.377 (3)	C16—H16B	0.9700
C2—H2	0.9300	C17—C18	1.519 (2)
C3—C4	1.378 (3)	C17—H17A	0.9700
C3—H3	0.9300	C17—H17B	0.9700
C4—C5	1.377 (3)	C18—C19	1.517 (3)
C4—H4	0.9300	C18—H18A	0.9700
C5—C6	1.398 (2)	C18—H18B	0.9700
C5—H5	0.9300	C19—C20	1.521 (2)
C7—H7A	0.9600	C19—H19A	0.9700
C7—H7B	0.9600	C19—H19B	0.9700
C7—H7C	0.9600	C20—C21	1.515 (3)
C8—C9	1.528 (2)	C20—H20A	0.9700
C9—C10	1.526 (2)	C20—H20B	0.9700
C9—C12	1.529 (2)	C21—C22	1.529 (2)
C9—H9	0.9800	C21—H21A	0.9700
C11—H11A	0.9600	C21—H21B	0.9700
C11—H11B	0.9600	C22—C23	1.497 (3)
C11—H11C	0.9600	C22—H22A	0.9700
C12—C13	1.516 (3)	C22—H22B	0.9700
C12—H12A	0.9700	C23—H23A	0.9700
C12—H12B	0.9700	C23—H23B	0.9700
			
C8—N1—C1	123.32 (14)	C15—C14—C13	113.01 (14)
C8—N1—C7	118.69 (14)	C15—C14—H14A	109.0
C1—N1—C7	117.93 (13)	C13—C14—H14A	109.0
C10—N2—C6	123.20 (14)	C15—C14—H14B	109.0
C10—N2—C11	117.90 (14)	C13—C14—H14B	109.0
C6—N2—C11	118.61 (13)	H14A—C14—H14B	107.8
C6—C1—C2	119.76 (15)	C14—C15—C16	114.23 (14)
C6—C1—N1	121.61 (15)	C14—C15—H15A	108.7
C2—C1—N1	118.59 (15)	C16—C15—H15A	108.7
C3—C2—C1	120.02 (17)	C14—C15—H15B	108.7
C3—C2—H2	120.0	C16—C15—H15B	108.7
C1—C2—H2	120.0	H15A—C15—H15B	107.6
C2—C3—C4	120.41 (17)	C17—C16—C15	113.12 (14)
C2—C3—H3	119.8	C17—C16—H16A	109.0
C4—C3—H3	119.8	C15—C16—H16A	109.0
C5—C4—C3	120.00 (17)	C17—C16—H16B	109.0
C5—C4—H4	120.0	C15—C16—H16B	109.0
C3—C4—H4	120.0	H16A—C16—H16B	107.8
C4—C5—C6	120.50 (17)	C16—C17—C18	114.08 (14)
C4—C5—H5	119.7	C16—C17—H17A	108.7
C6—C5—H5	119.7	C18—C17—H17A	108.7
C1—C6—C5	119.30 (15)	C16—C17—H17B	108.7
C1—C6—N2	121.91 (14)	C18—C17—H17B	108.7
C5—C6—N2	118.76 (15)	H17A—C17—H17B	107.6
N1—C7—H7A	109.5	C19—C18—C17	113.72 (15)
N1—C7—H7B	109.5	C19—C18—H18A	108.8
H7A—C7—H7B	109.5	C17—C18—H18A	108.8
N1—C7—H7C	109.5	C19—C18—H18B	108.8
H7A—C7—H7C	109.5	C17—C18—H18B	108.8
H7B—C7—H7C	109.5	H18A—C18—H18B	107.7
O1—C8—N1	122.01 (16)	C18—C19—C20	114.64 (15)
O1—C8—C9	122.12 (15)	C18—C19—H19A	108.6
N1—C8—C9	115.85 (14)	C20—C19—H19A	108.6
C10—C9—C8	106.92 (14)	C18—C19—H19B	108.6
C10—C9—C12	112.15 (14)	C20—C19—H19B	108.6
C8—C9—C12	111.47 (14)	H19A—C19—H19B	107.6
C10—C9—H9	108.7	C21—C20—C19	112.58 (15)
C8—C9—H9	108.7	C21—C20—H20A	109.1
C12—C9—H9	108.7	C19—C20—H20A	109.1
O2—C10—N2	121.92 (16)	C21—C20—H20B	109.1
O2—C10—C9	122.23 (15)	C19—C20—H20B	109.1
N2—C10—C9	115.83 (14)	H20A—C20—H20B	107.8
N2—C11—H11A	109.5	C20—C21—C22	114.90 (15)
N2—C11—H11B	109.5	C20—C21—H21A	108.5
H11A—C11—H11B	109.5	C22—C21—H21A	108.5
N2—C11—H11C	109.5	C20—C21—H21B	108.5
H11A—C11—H11C	109.5	C22—C21—H21B	108.5
H11B—C11—H11C	109.5	H21A—C21—H21B	107.5
C13—C12—C9	111.55 (14)	C23—C22—C21	109.44 (14)
C13—C12—H12A	109.3	C23—C22—H22A	109.8
C9—C12—H12A	109.3	C21—C22—H22A	109.8
C13—C12—H12B	109.3	C23—C22—H22B	109.8
C9—C12—H12B	109.3	C21—C22—H22B	109.8
H12A—C12—H12B	108.0	H22A—C22—H22B	108.2
C12—C13—C14	114.09 (14)	C22—C23—Br1	114.43 (12)
C12—C13—H13A	108.7	C22—C23—H23A	108.7
C14—C13—H13A	108.7	Br1—C23—H23A	108.7
C12—C13—H13B	108.7	C22—C23—H23B	108.7
C14—C13—H13B	108.7	Br1—C23—H23B	108.7
H13A—C13—H13B	107.6	H23A—C23—H23B	107.6
			
C8—N1—C1—C6	50.6 (2)	N1—C8—C9—C10	−70.41 (18)
C7—N1—C1—C6	−132.37 (17)	O1—C8—C9—C12	−15.1 (2)
C8—N1—C1—C2	−131.72 (18)	N1—C8—C9—C12	166.69 (15)
C7—N1—C1—C2	45.3 (2)	C6—N2—C10—O2	−171.56 (16)
C6—C1—C2—C3	0.6 (3)	C11—N2—C10—O2	2.2 (3)
N1—C1—C2—C3	−177.09 (17)	C6—N2—C10—C9	9.8 (2)
C1—C2—C3—C4	0.3 (3)	C11—N2—C10—C9	−176.45 (15)
C2—C3—C4—C5	−1.1 (3)	C8—C9—C10—O2	−111.04 (18)
C3—C4—C5—C6	1.0 (3)	C12—C9—C10—O2	11.4 (2)
C2—C1—C6—C5	−0.7 (3)	C8—C9—C10—N2	67.61 (18)
N1—C1—C6—C5	176.96 (16)	C12—C9—C10—N2	−169.93 (15)
C2—C1—C6—N2	−178.75 (16)	C10—C9—C12—C13	164.64 (15)
N1—C1—C6—N2	−1.1 (3)	C8—C9—C12—C13	−75.51 (19)
C4—C5—C6—C1	−0.1 (3)	C9—C12—C13—C14	175.98 (15)
C4—C5—C6—N2	178.02 (17)	C12—C13—C14—C15	178.16 (16)
C10—N2—C6—C1	−51.4 (2)	C13—C14—C15—C16	175.04 (15)
C11—N2—C6—C1	134.90 (17)	C14—C15—C16—C17	178.80 (16)
C10—N2—C6—C5	130.52 (18)	C15—C16—C17—C18	−179.03 (16)
C11—N2—C6—C5	−43.2 (2)	C16—C17—C18—C19	178.06 (17)
C1—N1—C8—O1	176.05 (16)	C17—C18—C19—C20	−179.30 (17)
C7—N1—C8—O1	−0.9 (3)	C18—C19—C20—C21	−178.30 (16)
C1—N1—C8—C9	−5.7 (2)	C19—C20—C21—C22	175.93 (17)
C7—N1—C8—C9	177.33 (15)	C20—C21—C22—C23	−177.00 (16)
O1—C8—C9—C10	107.82 (18)	C21—C22—C23—Br1	173.24 (13)

### Hydrogen-bond geometry (Å, °)

**Table:**

*D*—H···*A*	*D*—H	H···*A*	*D*···*A*	*D*—H···*A*
C2—H2···O1^i^	0.93	2.52	3.424 (2)	164
C7—H7B···O2^ii^	0.96	2.40	3.340 (2)	166
C11—H11C···O1^ii^	0.96	2.48	3.407 (2)	164

Symmetry codes: (i) −*x*+1/2, *y*−1/2, −*z*+1/2; (ii) −*x*−1/2, *y*−1/2, −*z*+1/2.
